# Threat assessment shapes neutrophil cell fate upon inflammasome activation

**DOI:** 10.1126/sciadv.aeb4830

**Published:** 2026-06-26

**Authors:** See Jie Yow, Bing-Chen Wu, Hai Shin Pung, Safwah Nasuha Rosli, Hui Wen Yeap, Ghin Ray Goh, Kay En Low, Isabelle Bonne, Jizhong Shi, Shuizhou Yu, Motomi Osato, Davey Kneafsey, Gabrielle Chappell, Ye Chean Teh, Shu Zhen Chong, Melissa S. F. Ng, Immanuel Kwok, Jelena S. Bezbradica, Dave Boucher, Kaiwen W. Chen

**Affiliations:** ^1^ Immunology Translational Research Programme, Life Sciences Institute, National University of Singapore, Singapore 117456, Singapore.; ^2^ Department of Microbiology and Immunology, Yong Loo Lin School of Medicine, National University of Singapore, Singapore, Singapore.; ^3^ Electron Microscopy Unit, Microscopy Cluster, Yong Loo Lin School of Medicine, National University of Singapore, Singapore, Singapore.; ^4^ Transgenic and Gene Targeting Facility, Cancer Science Institute of Singapore, National University of Singapore, Singapore, Singapore.; ^5^ York Biomedical Research Institute and Department of Biology, University of York, York, UK.; ^6^ The Kennedy Institute of Rheumatology, University of Oxford, Oxford, UK.; ^7^ Singapore Immunology Network (SIgN), Agency for Science, Technology and Research (A*STAR), Singapore, Singapore.

## Abstract

Inflammasomes are cytosolic multiprotein complexes that activate caspase-1, which promotes inflammation and host defense by driving cytokine maturation and pyroptosis. Several studies reported that caspase-1 selectively drives cytokine maturation without concomitant pyroptosis in neutrophils, yet the molecular mechanisms by which neutrophils resist caspase-1–dependent pyroptosis remain unclear. Here, we report that granulocyte-macrophage colony-stimulating factor (GM-CSF) licenses neutrophil pyroptosis upon NLRP3 and Pyrin activation by amplifying TLR4-driven inflammasome priming. Single priming with the TLR1/2 agonist, Pam3CSK4, was also sufficient to license neutrophils to pyroptosis upon NLRP3 and Pyrin activation, as Pam3CSK4 triggered superior inflammasome priming compared to LPS, the prototypic inflammasome priming agent. We further demonstrate that neutrophil pyroptosis requires autocrine TNFR1 signaling and provides genetic evidence that *Ninj1^K45Q/K45Q^* mutation disrupts plasma membrane rupture in pyroptotic neutrophils. In contrast, NLRC4 expression was not further induced by GM-CSF and therefore does not enhance susceptibility to NLRC4-dependent pyroptosis. Collectively, our data demonstrate that the inflammatory environment dictates neutrophil cell fate upon inflammasome activation.

## Teaser

Inflammatory environment and TLR priming dictate neutrophil susceptibility to pyroptosis.

## INTRODUCTION

Innate immune cells express a suite of pattern recognition receptors to detect host- and pathogen-derived danger signals and initiate complex signal transduction pathways to restore homeostasis (*1*). Emerging studies demonstrate that the local tissue and inflammatory environment, including cytokines and metabolites, substantially influences myeloid cell function (*2*–*5*). However, whether inflammatory cytokines regulate inflammasome signaling in neutrophils remains poorly characterized.

Inflammasomes are multiprotein complexes that assemble in the cytosol following microbial infection or cellular stress (*6*, *7*). Inflammasomes comprise a sensor protein, such as nucleotide-binding domain and leucine-rich repeat-containing pyrin protein 3 (NLRP3), NLR family CARD domain-containing protein 4 (NLRC4), and Pyrin, and are connected to the effector protease caspase-1 via an adaptor protein, apoptosis-associated speck-like protein containing a CARD (ASC). NLRP3 is activated by a broad range of host and microbial signals that culminate in cellular stress (*8*); Pyrin senses RhoA inactivation (*9*); and NLRC4 detects bacterial flagellin and rod protein from the type 3 secretion system (T3SS) (*10*–*12*). Upon activation, the inflammasome sensor oligomerizes and recruits ASC to form a large filamentous structure that initiates caspase-1 recruitment, dimerization, and proximity-induced auto-activation (*13*). Active caspase-1 cleaves the precursor cytokines pro–interleukin-1β (IL-1β) and IL-18 into its mature biologically active fragment and initiates calpain-dependent pro–IL-1α processing (*7*, *14*, *15*). Caspase-1 also cleaves gasdermin D (GSDMD) to release the cytotoxic N-terminal fragment that inserts into the inner leaflet of the plasma membrane to drive a form of lytic cell death termed “pyroptosis” (*16*–*18*). Recent studies further revealed that oligomerization of the plasma membrane protein, Ninjurin-1 (NINJ1), triggers large plasma membrane lesions to promote the final plasma membrane rupture (PMR) upon inflammasome activation (*19*–*21*). Because GSDMD pores serve as a conduit for cytokine release from living cells (*22*–*24*), *Ninj1* deficiency suppresses PMR but does not impair IL-1α, IL-1β, and IL-18 release from inflammasome-activated cells (*19*).

Neutrophils are one of the first cell types to be recruited in large quantities to the site of infection or tissue injury and play a critical role in protecting the mammalian host from infection (*25*). Unlike other myeloid cells, neutrophils have a short life span ranging from 1 to 2 days (*26*, *27*). However, during infection and disease, microbial components or proinflammatory cytokines such as granulocyte colony-stimulating factor (G-CSF), granulocyte-macrophage colony-stimulating factor (GM-CSF), and interferon-γ (IFN-γ) in the tissue environment suppress neutrophil cell death and extend their life span to enable these cells to execute their antimicrobial functions (*27*–*31*). In agreement with this, we and others previously demonstrated that activation of the NLRC4, NLRP3, and Pyrin inflammasome in neutrophils selectively promotes IL-1β secretion without triggering pyroptosis (*32*–*37*). This unique property enables neutrophils to perform their antimicrobial functions, sustain IL-1β release, and serve as a major cellular source of IL-1β during infection (*33*, *38*). However, despite more than one decade of research, the molecular mechanisms by which neutrophils resist caspase-1–dependent pyroptosis still remain poorly understood. One study proposed that cleaved GSDMD preferentially traffics toward azurophilic granules and LC3^+^ autophagosome to reduce plasma membrane damage (*35*), while another study reported that delivery of bacterial effectors and toxins from the endosomes switches off the pyroptotic program in neutrophils (*36*). In contrast, we found that neutrophils express low levels of ASC and proposed that this restricts neutrophil caspase-1 activity to sublethal levels (*13*, *34*).

Here, we report that TLR signaling and the cytokine environment dynamically regulate neutrophil susceptibility to NLRP3 and Pyrin-dependent pyroptosis. Mechanistically, the inflammatory cytokines GM-CSF and, to a lesser extent, IFN-γ enhance lipopolysaccharide (LPS)-induced NLRP3 and Pyrin expression, leading to enhanced caspase-1 activation, GSDMD cleavage, and licensed NINJ1-dependent PMR upon inflammasome activation. The TLR1/2 agonist, Pam3CSK4, also triggered stronger NLRP3 and Pyrin expression compared to LPS and, by itself, is sufficient to trigger GSDMD-dependent pyroptosis upon NLRP3 and Pyrin inflammasome activation. Like LPS priming, GM-CSF and, to a lesser extent, IFN-γ further enhanced Pam3CSK4-induced NLRP3 and Pyrin expression, inflammasome activation, and pyroptosis. Conversely, NLRC4 expression remained similar upon LPS and Pam3CSK4 priming and was not further induced by GM-CSF or IFN-γ; therefore, these inflammatory cytokines do not enhance the susceptibility to pyroptosis downstream of NLRC4 activation. Collectively, our data demonstrate that the inflammatory environment and the type of threat dictate neutrophil death fate upon inflammasome activation.

## RESULTS

### GM-CSF licenses LPS-primed neutrophils to pyroptosis upon NLRP3 activation

**Fig. 1.**
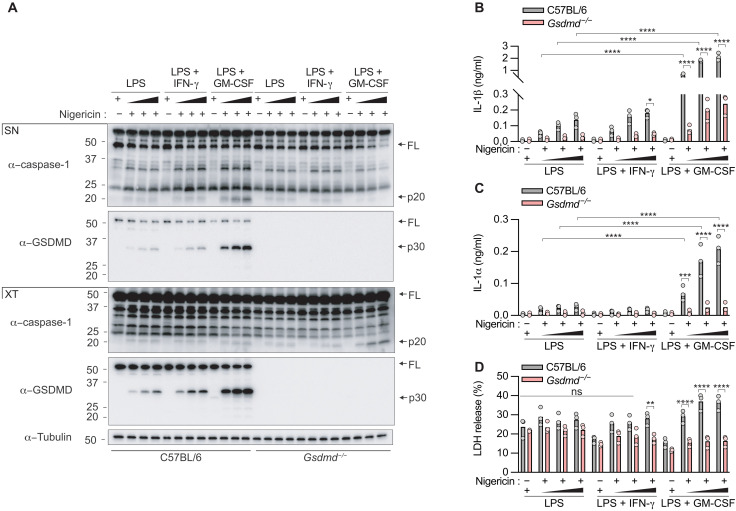
GM-CSF licenses LPS-primed neutrophils to pyroptosis upon NLRP3 activation. (**A** to **D**) Neutrophils were primed with increasing dose of ultrapure LPS (0.1, 0.5, and 1 μg/ml) in the presence or absence of IFN-γ (10, 50, and 100 ng/ml) or GM-CSF (10, 50, and 100 ng/ml) for 4 hours and stimulated with nigericin (5 μM) for 2 hours. (A) Precipitated supernatant (SN) and cell extracts (XT) were analyzed by immunoblot. (B) IL-1β, (C) IL-1α, and (D) LDH release were quantified. [(B) to (D)] Data represent mean value pooled from three independent experiments. **P < 0.01, ***P < 0.001, and ****P < 0.0001; ns, not significant.

We previously demonstrated that the proinflammatory cytokine, IFN-γ, licenses *Yersinia-*infected neutrophils to trigger GSDME-dependent pyroptosis, a closely related member of the GSDMD protein family (*27*, *31*, *39*). This prompted us to investigate whether IFN-γ or other proinflammatory cytokines such as GM-CSF that are often elevated during inflammation similarly sensitize neutrophils to GSDMD-dependent pyroptosis downstream of canonical inflammasome activation. NLRP3 activation typically requires TLR priming by TLR ligands to up-regulate NLRP3 and pro–IL-1 expression (*40*), followed by stimulation with a second threat signal, such as the bacterial ionophore, nigericin, to trigger NLRP3 complex assembly. To mimic an inflammatory environment, we purified bone marrow neutrophils from wild-type (WT) or *Gsdmd^−/−^* mice using positive selection as previously described (*27*, *29*, *33*, *34*, *41*) and primed them with the TLR4 ligand, LPS, in the presence or absence of IFN-γ or GM-CSF, before stimulating these cells with nigericin to activate the NLRP3 inflammasome. In agreement with previous reports (*34*, *35*, *42*), nigericin stimulation on LPS-primed neutrophils triggered caspase-1 processing (Fig. 1A), GSDMD cleavage (Fig. 1A), IL-1β secretion (Fig. 1B), but not IL-1α release (Fig. 1C) or GSDMD-dependent lactate dehydrogenase (LDH) release (Fig. 1D), a measurement of lytic cell death. IFN-γ and LPS copriming had minimal impact on nigericin-induced IL-1 secretion (Fig. 1, B and C) but modestly increased caspase-1 and GSDMD processing compared to LPS-primed neutrophils (Fig. 1A) and sensitized neutrophils to GSDMD-dependent cell death at the highest dose examined (Fig. 1D). Excitingly, LPS and GM-CSF copriming strongly enhanced nigericin-induced caspase-1 and GSDMD cleavage compared to LPS-primed neutrophils (Fig. 1A and fig. S1, A to C), sensitized neutrophils to robust GSDMD-dependent pyroptosis at all concentration examined (Fig. 1D), and induced 10- to 15-fold higher IL-1α/β secretion compared to LPS-primed neutrophils (Fig. 1, B and C). In contrast, GM-CSF priming by itself was unable to license pyroptosis (fig. S1D) or IL-1β secretion (fig. S1E) upon nigericin stimulation. G-CSF that is often associated with neutrophil activation (*43*) had minimal impact on nigericin-induced pyroptosis and cytokine secretion compared to LPS-primed neutrophils (fig. S1, F to H). We further confirmed that IFN-γ and GM-CSF also enhanced nigericin-induced pyroptosis and IL-1α/β secretion from negatively selected LPS-primed bone marrow neutrophils (fig. S1, I to L). Together, these data demonstrate that GM-CSF and IFN-γ, to a lesser extent, license LPS-primed neutrophils to NLRP3- and GSDMD-dependent pyroptosis.

### TLR2 priming licenses neutrophil pyroptosis upon NLRP3 activation

**Fig. 2.**
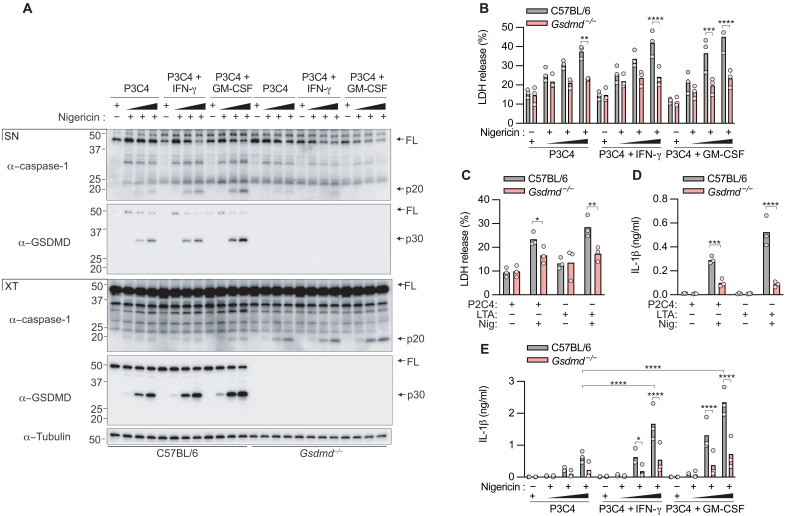
TLR2 priming licenses neutrophil pyroptosis upon NLRP3 activation. (**A**, **B**, and **E**) Neutrophils were primed with increasing dose of Pam3CSK4 (P3C4; 0.1, 0.5, and 1 μg/ml) in the presence or absence of IFN-γ (10, 50, and 100 ng/ml) or GM-CSF (10, 50, and 100 ng/ml) for 4 hours and stimulated with nigericin (5 μM) for 2 hours. (**C** and **D**) Neutrophils were primed with Pam2CSK4 (P2C4; 1 μg/ml) or lipoteichoic acid (LTA; 1 μg/ml) for 4 hours and stimulated with nigericin (5 μM) for 2 hours. (A) Precipitated supernatant (SN) and cell extracts (XT) were analyzed by immunoblot. [(B) and (C)] LDH and [(D) and (E)] cytokine release were quantified. [(B) to (D)] Data represent mean value pooled from three independent experiments. *P < 0.05, **P < 0.01, ***P < 0.001, and ****P < 0.0001.

To determine whether the synergistic effect of LPS and IFN-γ or GM-CSF copriming on NLRP3 activation is restricted to TLR4 signaling, we primed neutrophils with the synthetic TLR1/2 agonist, Pam3CSK4, in the presence or absence of IFN-γ or GM-CSF and exposed them to nigericin. As anticipated, nigericin triggered caspase-1 and GSDMD cleavage in Pam3CSK4-primed neutrophils (Fig. 2A). Unexpectedly, unlike in LPS-primed conditions (Fig. 1D), Pam3CSK4 priming at the highest dose is sufficient to license GSDMD-dependent neutrophil pyroptosis upon nigericin stimulation (Fig. 2B). Priming neutrophils with other TLR2 ligands such as Pam2CSK4 or lipoteichoic acid (LTA) was also sufficient to sensitize neutrophils to pyroptosis and IL-1β secretion upon nigericin stimulation (Fig. 2, C and D). Consistent with LPS-primed conditions (Fig. 1), GM-CSF further amplified caspase-1 processing (Fig. 2A and fig. S2A), GSDMD cleavage (Fig. 2A and fig. S2, B and C), pyroptosis (Fig. 2B), and IL-1α/β secretion (Fig. 2E and fig. S2D) upon nigericin stimulation in Pam3CSK4-primed neutrophils.

### NINJ1 oligomerization promotes neutrophil PMR

**Fig. 3.**
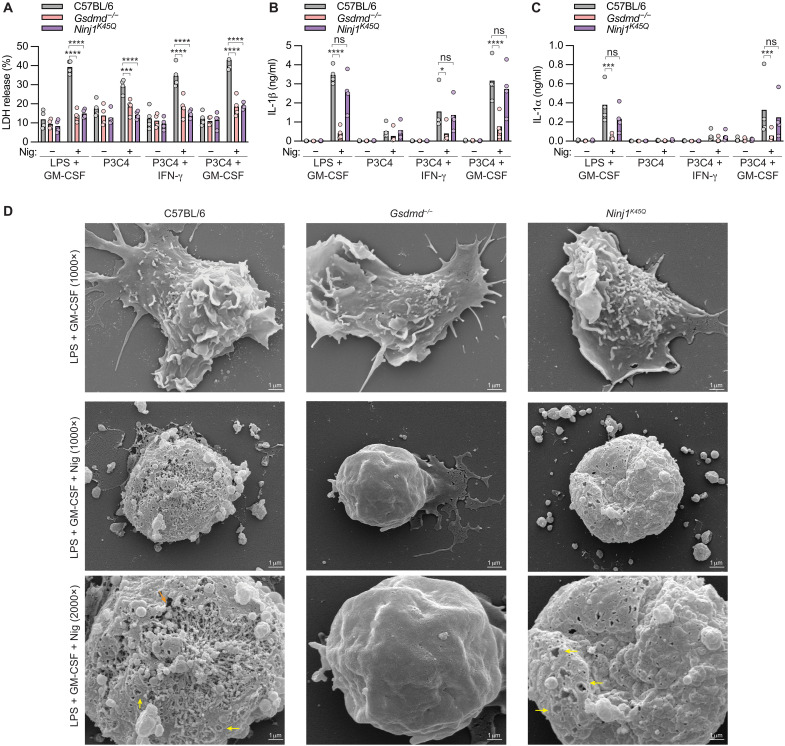
NINJ1 oligomerization promotes neutrophil plasma membrane rupture. (**A** to **C**) Neutrophils were primed with ultrapure LPS (1 μg/ml) or Pam3CSK4 (P3C4; 1 μg/ml) in the presence or absence of IFN-γ (100 ng/ml) or GM-CSF (100 ng/ml) for 4 hours and stimulated with nigericin (5 μM) for 2 hours. (A) LDH and [(B) and (C)] IL-1 release were quantified. (**D**) Scanning electron microscopy images of inflammasome-activated neutrophils. The orange arrow denotes large membrane pores (>1 μM) while yellow arrows denote small membrane pores (<1 μM). [(A) to (C)] Data represent mean value pooled from three independent experiments. *P < 0.05, ***P < 0.001, and ****P < 0.0001.

To examine whether neutrophil PMR requires NINJ1 oligomerization, we generated a *Ninj1* knock-in mouse in which we mutated amino acid lysine-45 to glutamine (*Ninj1^K45Q^*) to impair NINJ1 oligomerization (*19*). Unlike *Ninj1*^−/−^ animals that develop microencephaly (*44*), homozygous *Ninj1^K45Q^* mice are fertile and healthy and do not display any overt developmental abnormalities (fig. S3, A to G). Although nigericin triggered comparable caspase-1 and GSDMD cleavage between WT and *Ninj1^K45Q^* neutrophils (fig. S3H), *Ninj1^K45Q^* mutation reduced nigericin-induced LDH release to the same levels seen in *Gsdmd^−/−^* neutrophils (Fig. 3A), indicating that NINJ1 oligomerization is required for the final PMR downstream of canonical inflammasome activation in neutrophils. WT and *Ninj1^K45Q^* neutrophils displayed comparable IL-1α/β secretion (Fig. 3, B and C), consistent with the ability of GSDMD pores to mediate IL-1α/β release in the absence of PMR (*19*, *22*–*24*).

To further characterize the morphological features of pyroptotic neutrophils, we stimulated LPS and GM-CSF coprimed neutrophils with nigericin to induce GSDMD- and NINJ1-dependent pyroptosis and performed scanning electron microscopy (SEM). Nigericin triggered rounding and cellular shrinkage in LPS and GM-CSF coprimed WT, *Gsdmd^−/−^*, and *Ninj1^K45Q^* neutrophils; however, the formation of membrane blebs was only observed in WT and *Ninj1^K45Q^* neutrophils (Fig. 3D and fig. S3I). Pyroptotic WT neutrophils displayed circular membrane pores of varying sizes ranging from several nanometers (yellow arrows) to micrometers (orange arrows), while the membrane appears largely intact in *Gsdmd^−/−^* neutrophils following nigericin stimulation (Fig. 3D and fig. S3I). Nucleic acid structures were contained beneath membrane pores in pyroptotic WT neutrophils (Fig. 3D and fig. S3I), indicating that unlike the noncanonical caspase-4/5/11 inflammasome activation by cytosolic LPS (*34*, *45*), canonical NLRP3 and caspase-1 activation does not induce the release of neutrophil extracellular traps, consistent with a previous report (*37*). Although *Ninj1^K45Q^* neutrophils are resistant to LDH release and PMR (Fig. 3A), smaller pores in the nanometer range were observed on the plasma membrane (Fig. 3D and fig. S3I), which correlate broadly within the reported diameter of GSDMD pores (*39*, *46*–*48*). Similar results were obtained when Pam3CSK4-primed and Pam3CSK4 and GM-CSF coprimed neutrophils were stimulated with nigericin (fig. S4, A to D).

### GM-CSF enhances LPS-induced inflammasome priming while TLR2 agonists elicit stronger NLRP3 expression than LPS

**Fig. 4.**
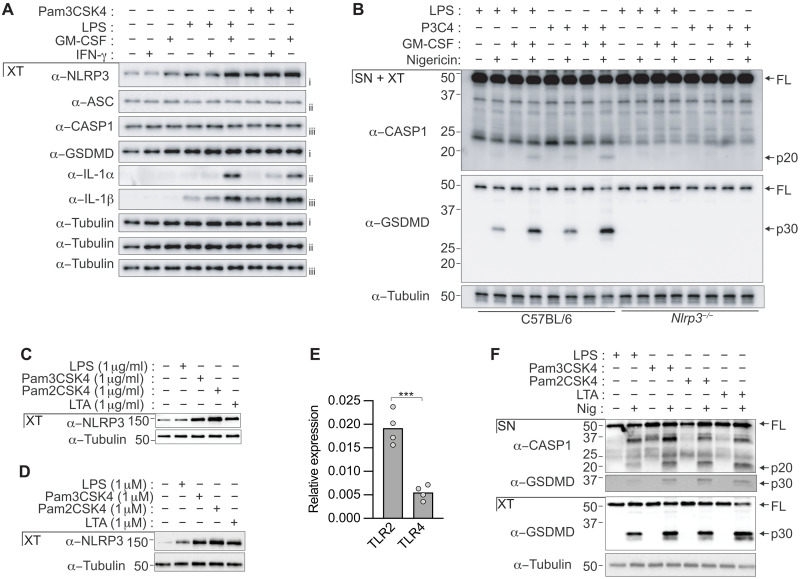
GM-CSF enhances LPS-induced inflammasome priming while TLR2 agonists elicit stronger NLRP3 expression than LPS. (**A**) Neutrophils were primed with various combinations of ultrapure LPS (1 μg/ml), Pam3CSK4 (P3C4; 1 μg/ml), IFN-γ (100 ng/ml), or GM-CSF (100 ng/ml) for 4 hours, and cell extracts were analyzed by immunoblotting. (**B**) Neutrophils were primed with ultrapure LPS (1 μg/ml) or Pam3CSK4 (P3C4; 1 μg/ml) in the presence or absence of GM-CSF (100 ng/ml) for 4 hours and stimulated with nigericin (5 μM) for 2 hours. Molarity was calculated using an estimated molecular weight for LPS (855.4 g/mol) and LTA (775 g/mol), and a defined molecular weight for Pam2CSK4 (1271.85 g/mol) and Pam3CSK (1852.33 g/mol). Precipitated supernatant (SN) and cell extracts (XT) were analyzed by immunoblot. (**C** and **D**) Neutrophils were primed with either 1 μg/ml or 1 μM ultrapure LPS, Pam3CSK4, Pam2CSK4, or LTA for 4 hours, and NLRP3 expression was analyzed by immunoblot. (**E**) Neutrophil TLR2 and TLR4 expression were quantified by qPCR. (**F**) Neutrophils were primed with 1 μM ultrapure LPS, Pam3CSK4, Pam2CSK4, or LTA for 4 hours and stimulated with nigericin (5 μM) for 2 hours. SN and XT were analyzed by immunoblot. (E) Data represent mean value pooled from four independent experiments. ***P < 0.001.

To elucidate the mechanisms by which the different priming regimen sensitizes neutrophils to GSDMD-dependent pyroptosis, we examined the expression of individual NLRP3 signaling components before and after priming. Consistent with previous reports (*27*, *33*, *40*), LPS induced NLRP3 and pro–IL-1β expression compared to unstimulated neutrophils, while ASC, caspase-1, and GSDMD are constitutively expressed and not further induced upon LPS exposure (Fig. 4A and fig. S5, A and B). GM-CSF by itself induced modest NLRP3 expression (Fig. 4A and fig. S5, A and B), but strongly enhanced LPS-induced NLRP3 and pro–IL-1α/β expression (Fig. 4A and fig. S5, A and B). Consequently, GM-CSF enhanced NLRP3-dependent caspase-1 cleavage (Figs. 1A and 4B and fig. S1A), GSDMD processing (Figs. 1A and 4B and fig. S1, B and C), LDH release (Fig. 1D), and IL-1 secretion (Fig. 1, C and D) following nigericin stimulation compared to LPS-primed neutrophils. Pam3CSK4, Pam2CSK4, and LTA at 1 μg/ml or 1 μM all triggered stronger NLRP3 expression than LPS (Fig. 4, C and D, and fig. S5, C and D), possibly due to higher neutrophil TLR2 expression compared to TLR4 (Fig. 4E). Consequently, TLR2-primed neutrophils displayed stronger nigericin-induced caspase-1 and GSDMD processing compared to LPS-primed neutrophils (Fig. 4F and fig. S5, E and F), explaining why Pam3CSK4, Pam2CSK4, and LTA (Fig. 2, B and C) but not LPS priming alone (Fig. 1D) licenses neutrophil pyroptosis upon NLRP3 activation.

### NLRP3-dependent neutrophil pyroptosis requires TNFR1 signaling

**Fig. 5.**
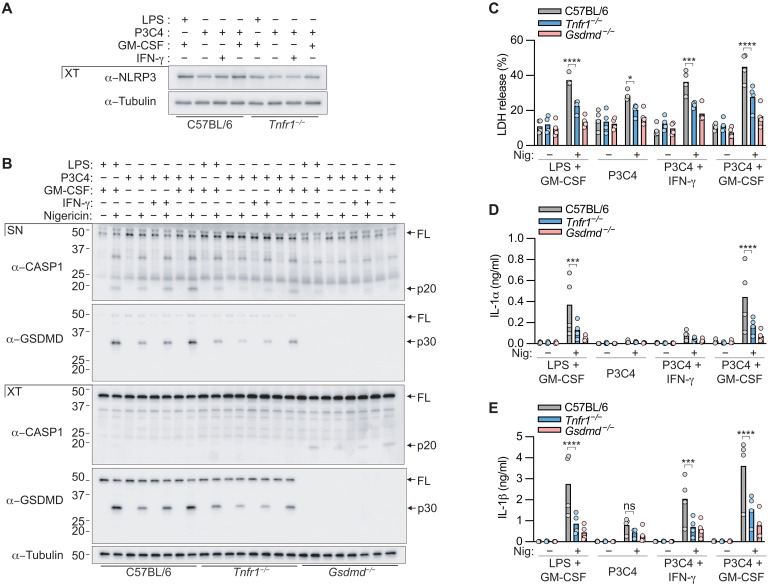
NLRP3-dependent neutrophil pyroptosis requires TNFR1 signaling. (**A**) Neutrophils were primed with various combinations of ultrapure LPS (1 μg/ml), Pam3CSK4 (P3C4; 1 μg/ml), IFN-γ (100 ng/ml), or GM-CSF (100 ng/ml) for 4 hours, and cell extracts were analyzed by immunoblotting. (**B** to **E**) Neutrophils were primed with ultrapure LPS (1 μg/ml) or Pam3CSK4 (P3C4; 1 μg/ml) in the presence or absence of IFN-γ (100 ng/ml) or GM-CSF (100 ng/ml) for 4 hours and stimulated with nigericin (5 μM) for 2 hours. (B) Mixed supernatant (SN) and cell extracts (XT) were analyzed by immunoblot. (C) LDH and [(D) and (E)] cytokine release were quantified. [(C) to (E)] Data represent mean value pooled from four independent experiments. *P < 0.05, ***P < 0.001, and ****P < 0.0001.

We and others previously demonstrated that autocrine tumor necrosis factor receptor 1 (TNFR1) signaling enhances expression of TLR-induced cytokines in neutrophils (*49*), including the inflammasome substrate, pro–IL-1β (*31*). Thus, we examined whether autocrine TNFR1 signaling likewise promotes TLR and cytokine-induced neutrophil NLRP3 expression and its susceptibility to GSDMD-dependent pyroptosis. *Tnfr1* deficiency impaired TLR and cytokine-induced NLRP3 expression (Fig. 5A and fig. S6A) and nigericin-induced caspase-1 and GSDMD cleavage compared to WT neutrophils (Fig. 5B and fig. S6, B and C). Consequently, *Tnfr1* deficiency significantly reduced nigericin-induced pyroptosis (Fig. 5C) and IL-1 secretion (Fig. 5, D and E) compared to WT neutrophils, although not to the same extent as *Gsdmd^−/−^* neutrophils.

### GM-CSF licenses LPS-primed neutrophils to pyroptosis upon pyrin activation

**Fig. 6.**
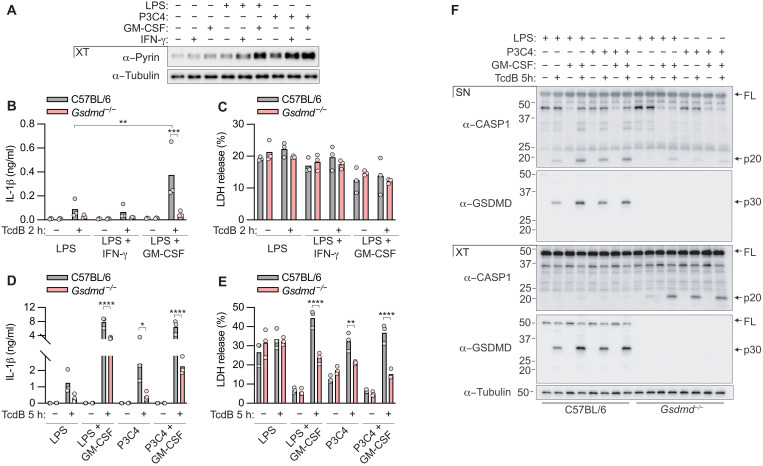
GM-CSF licenses LPS-primed neutrophils to pyroptosis upon Pyrin activation. (**A**) Neutrophils were primed with various combinations of ultrapure LPS (1 μg/ml), Pam3CSK4 (P3C4; 1 μg/ml), IFN-γ (100 ng/ml), or GM-CSF (100 ng/ml) for 4 hours, and cell extracts were analyzed by immunoblotting. (**B** to **F**) Neutrophils were primed with ultrapure LPS (1 μg/ml) or Pam3CSK4 (P3C4; 1 μg/ml) in the presence or absence of IFN-γ (100 ng/ml) or GM-CSF (100 ng/ml) for 4 hours and stimulated with TcdB (1 μg/ml) for [(B) and (C)] 2 hours or [(D) to (F)] 5 hours. (F) Precipitated supernatant (SN) and cell extracts (XT) were analyzed by immunoblot. [(B) and (D)] IL-1β and [(C) and (E)] LDH release were quantified. [(B) to (E)] Data represent mean value pooled from three independent experiments. *P < 0.05, **P < 0.01, ***P < 0.001, and ****P < 0.0001.

To determine whether TLR and cytokine priming licenses neutrophil pyroptosis downstream of other canonical inflammasomes, we primed neutrophils with the same priming regimen as above (Fig. 4A) and examined Pyrin expression (Fig. 6A). Consistent with our observations for NLRP3 (Fig. 4A), Pam3CSK4 induced stronger Pyrin expression compared to LPS (Fig. 6A and fig. S7A), but in both cases, the addition of GM-CSF and IFN-γ, to a lesser extent, further enhanced the TLR-induced Pyrin expression.

To examine whether GM-CSF or IFN-γ similarly licenses LPS-primed neutrophils to Pyrin-dependent pyroptosis as observed for the NLRP3 inflammasome (Fig. 1, A and D), we primed neutrophils with LPS in the presence or absence of IFN-γ or GM-CSF and exposed these cells to recombinant TcdB for 2 hours to activate the Pyrin inflammasome (*9*, *50*). Consistent with a previous report (*36*), TcdB triggered caspase-1 processing, GSDMD cleavage (fig. S7B), and IL-1β secretion (Fig. 6B) but not GSDMD-dependent pyroptosis in LPS-primed neutrophils (Fig. 6C). IFN-γ modestly enhanced LPS-induced Pyrin expression (Fig. 6A and fig. S7A) but did not further promote caspase-1 processing, GSDMD cleavage (fig. S7B), IL-1β secretion, and pyroptosis compared to LPS-primed neutrophils upon TcdB stimulation (Fig. 6, B and C). In contrast, GM-CSF strongly enhanced LPS-induced Pyrin expression (Fig. 6A and fig. S7A), and consequently amplified TcdB-induced caspase-1 processing and GSDMD cleavage (fig. S7B) and triggered fourfold higher IL-1β release compared to LPS-primed neutrophils (Fig. 6B). Unexpectedly, despite observing robust GSDMD cleavage in LPS and GM-CSF coprimed neutrophils following TcdB stimulation (fig. S7B), LDH release remains comparable between WT and *Gsdmd^−/−^* neutrophils at 2 hours post-TcdB treatment (Fig. 6C). To examine whether prolonged TcdB stimulation would create sufficient GSDMD pores to trigger pyroptosis, we primed WT and *Gsdmd^−/−^* neutrophils with LPS or Pam3CSK4 in the presence or absence of GM-CSF to represent varying levels of Pyrin expression (Fig. 6A and fig. S7A) and stimulated these cells with TcdB for 5 hours. Although increasing TcdB stimulation from 2 to 5 hours increased the amount of IL-1β release from LPS-primed neutrophils (Fig. 6, B and D), LDH release remains comparable between LPS-primed WT and *Gsdmd^−/−^* neutrophils at 5 hours post-TcdB stimulation (Fig. 6E), indicating that prolonged Pyrin activation does not elicit pyroptosis in LPS-primed neutrophils. In contrast, LPS and GM-CSF coprimed neutrophils that express high levels of Pyrin (Fig. 6A and fig. S7A) exhibited markedly enhanced caspase-1 processing and GSDMD cleavage compared to LPS-primed neutrophils (fig. S7, B to D) and displayed robust GSDMD-dependent pyroptosis and IL-1 secretion (Fig. 6, D and E). Pam3CSK4 or Pam3CSK4 and GM-CSF coprimed neutrophils that express higher Pyrin than LPS-primed neutrophils (Fig. 6A and fig. S7A) were also susceptible to GSDMD-dependent pyroptosis at 5 hours post-TcdB stimulation (Fig. 6E), as anticipated. We further confirmed that GM-CSF sensitizes negatively selected LPS-primed neutrophils to pyroptosis and IL-1 secretion upon 5 hours of TcdB stimulation (fig. S7, E to G). Together, these data demonstrate that it is the combination of enhanced Pyrin expression and TcdB stimulation duration that dictates neutrophil susceptibility to Pyrin-dependent pyroptosis.

### GM-CSF does not enhance NLRC4 expression and neutrophil pyroptosis upon NLRC4 activation

**Fig. 7.**
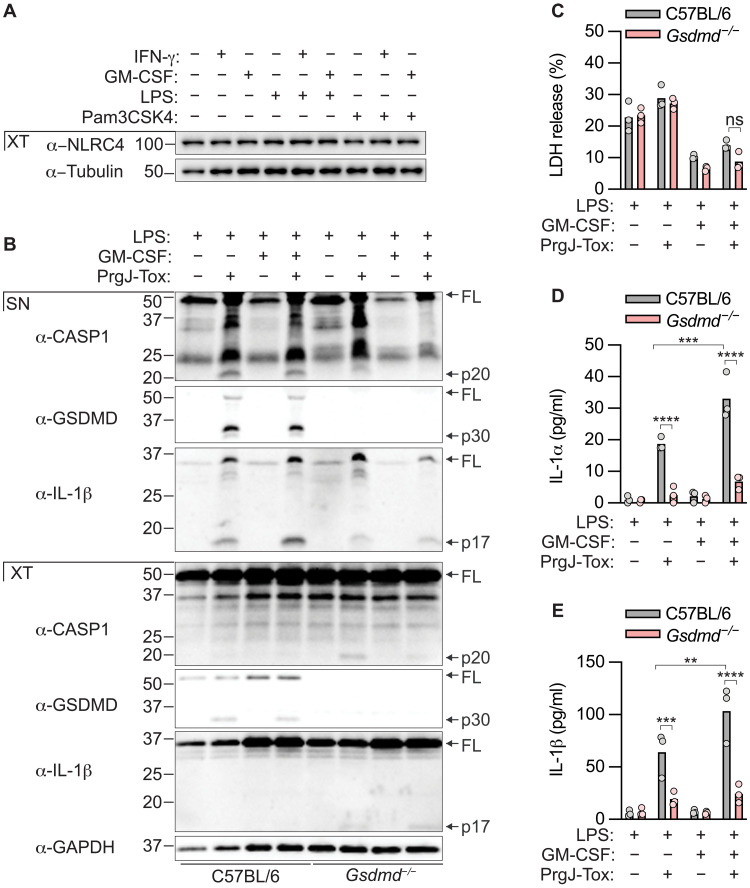
GM-CSF does not enhance NLRC4 expression and neutrophil pyroptosis upon NLRC4 activation. (**A**) Neutrophils were primed with various combinations of ultrapure LPS (1 μg/ml), Pam3CSK4 (P3C4; 1 μg/ml), IFN-γ (100 ng/ml), or GM-CSF (100 ng/ml) for 4 hours, and cell extracts were analyzed by immunoblotting. (**B** to **E**) Neutrophils were primed with ultrapure LPS (1 μg/ml) in the presence or absence of GM-CSF (100 ng/ml) for 4 hours and stimulated with PrgJ-Tox (2 μg/ml) for another 4 hours. (B) Precipitated supernatants (SN) and cell extracts (XT) were analyzed by immunoblots. (C) LDH, (D) IL-1α, and (E) IL-1β release were measured at 4 hours after PrgJ-Tox stimulation. [(C) to (E)] Data represent mean value pooled from three independent experiments. **P < 0.01, ***P < 0.001, and ****P < 0.0001.

Last, we observed that unlike NLRP3 (Fig. 4A) and Pyrin (Fig. 6A), neutrophil NLRC4 expression remains comparable under all conditions tested (Fig. 7A), which predicts that GM-CSF and LPS copriming should only enhance IL-1α/β secretion (Fig. 4A and fig. S5C), but not caspase-1 activation, GSDMD cleavage, and pyroptosis in NLRC4-stimulated neutrophils. To formally examine this, we primed WT and *Gsdmd^−/−^* neutrophils with LPS in the presence or absence of GM-CSF and stimulated the cells with PrgJ-Tox to mimic cytosolic infection and activate the NLRC4 inflammasome. Consistent with our prediction (Fig. 7A), GM-CSF did not enhance caspase-1 processing, GSDMD cleavage, and GSDMD-dependent cell death in LPS-primed neutrophils upon NLRC4 activation (Fig. 7, B and C) but only induced higher IL-1α/β secretion compared to LPS-primed neutrophils (Fig. 7, D and E).

## DISCUSSION

Programmed cell death such as pyroptosis and necroptosis are highly inflammatory antimicrobial mechanisms that destroy the replicative niche of intracellular pathogens and, thus, are often studied in cell types such as macrophages and epithelial cells that are susceptible to microbial replication. In contrast, neutrophils are professional phagocytes that are equipped with a suite of microbicidal features that are less permissive for pathogen survival, suggesting that the threshold for activating lytic cell death pathways may be higher in these cells. In agreement with this, neutrophils were reported to resist caspase-1 and GSDMD-dependent pyroptosis in response to a wide variety of inflammasome stimuli including the NLRP3 agonists nigericin (*34*, *35*) and ATP (*51*); Gram-negative bacteria such as *Salmonella* Typhimurium, *Legionella pneumoniae*, and *Burkholderia pseudomallei* that activate the NLRC4 inflammasome (*32*, *33*, *36*, *37*); and *Burkholderia cenocepacia* and TcdB that activate the Pyrin inflammasome (*36*). In contrast, detection of cytosolic LPS from Gram-negative bacteria triggers robust GSDMD-dependent neutrophil pyroptosis and host defense in vivo (*34*, *45*, *52*), suggesting that neutrophils are not intrinsically resistant to GSDMD pores, but only undergo pyroptosis under conditions of heightened microbial threat. Consistent with this model, we now report that inflammatory cytokines such as GM-CSF and, to a lesser extent, IFN-γ, which are elevated during microbial infection, license LPS-primed neutrophils to NLRP3 and Pyrin-dependent pyroptosis and to secrete 10-fold higher IL-1α/β upon inflammasome activation. Mechanistically, GM-CSF amplifies LPS-induced NLRP3, Pyrin, and pro–IL-1α/β expression, leading to stronger caspase-1 activation, GSDMD cleavage, and IL-1 release, and licenses NINJ1-driven PMR upon inflammasome activation (fig. S8). Because GM-CSF also promotes TLR signaling to enhance IL-1 release from inflammatory monocytes during *Legionella* infection (*53*), this suggests that inflammasome activity is dynamically regulated by the tissue environment, and neutrophil pyroptotic responses are likely fine-tuned by the amount of proinflammatory cytokines at the site of infection.

Unexpectedly, we observed that several TLR2 agonists including Pam2CSK4, Pam3CSK4, and LTA, but not LPS priming alone, were sufficient to license neutrophils to pyroptosis upon NLRP3 activation, as TLR2 agonists triggered superior inflammasome priming compared to LPS, the prototypic inflammasome priming agent (fig. S8). We speculate that low expression of TLR4, CD14, and TRIF likely explains the weak TLR4 signaling observed in neutrophils (*31*, *41*).

In contrast to NLRP3 and Pyrin, all priming conditions examined in this study did not enhance neutrophil NLRC4 expression; thus, we did not identify a priming regimen that sensitizes neutrophils to pyroptosis upon NLRC4 activation following cytosolic flagellin exposure. However, we do not exclude the possibility that other conditions that may enhance NLRC4 expression or enable heighten flagellin or rod protein translocation can indeed trigger NLRC4-dependent pyroptosis in neutrophils. For instance, a recent study reported that *Pseudomonas aeruginosa* infection triggers NLRC4-dependent pyroptosis in unprimed neutrophils (*37*), raising the possibility that *P. aeruginosa* translocates flagellin or rod proteins more efficiently than other pathogens that fail to trigger neutrophil NLRC4-dependent pyroptosis*.* Alternatively, pathogen blockade of phagocytosis (*36*, *37*), enhanced T3SS expression, or deficiency in key microbicidal mechanisms (*54*, *55*) may trigger sustained translocation of flagellin, rod proteins, and toxins into neutrophils, thereby bypassing the threshold of caspase-1 and GSDMD activation to induce pyroptosis.

Last, emerging studies demonstrate that heterogeneity in neutrophil development and activation states triggers specialized effector functions during infection and disease (*4*, *56*, *57*); thus, it is tempting to speculate that neutrophil subsets have differential susceptibility to pyroptosis and other programmed cell death pathways. Future studies characterizing inflammasome biology in neutrophil subsets during infection and disease will be a priority.

## METHODS

### Mice

C57BL/6J (strain no. 000664), *Gsdmd^−/−^* (strain no. 032410), *Tnfr1^−/−^* (strain no. 003242), and *Nlrp3^−/−^* (strain no. 021302) mice were purchased from the Jackson Laboratory and bred in specific pathogen-free animal facilities at the National University of Singapore. *Ninj1^K45Q/K45Q^* mice were generated by Transgenic and Gene Targeting Facility at the National University of Singapore using CRISPR-Cas9 technology. The crRNA (TGCCAACAAGAAGAGCGCTG) targeting exon 2 of *Ninj1* was used to induce double-stranded DNA break, and the single-stranded oligodeoxynucleotide (TTTGCGGAACCGGCCCATCAATGTAAACCATTATGCCAACAAGCAGAGCGCTGCCGAGAGCATGCTGGACATCGCGCTGCTCATGGCCAA CGCGTCGCAG) was used to induce the following mutation: an AAG>CAG mutation to affect the K>Q change and a silent G>C mutation to destroy the PAM sequence. Founder mice were genotyped by sequencing using the following primers: 5′-CAACAGAAGCCCTACCTACAG-3′ (F), 5′-GGTCCTACCCAGGAAGATGAG-3′ (R). Founder mice were backcrossed to C57BL/6 for three consecutive generations. All animal experiments were carried out in accordance with the protocols approved by the Institutional Animal Care and Use Committee of the National University of Singapore (license RBR24i-0014).

### Neutrophil inflammasome assays

Primary neutrophils were purified from murine bone marrow using α-Ly6G microbeads (no. 130-120-337, Miltenyi Biotec; positive selection) or the neutrophil isolation kit (no. 130-097-658, Miltenyi Biotec; negative selection) according to the manufacturer’s protocol. All experiments were performed using positively selected neutrophils, unless stated otherwise in the figure legends. Neutrophils were seeded at a density of 4 × 10^5^ cells per well in 200 μl of Opti-MEM (Thermo Fisher Scientific) and stimulated on the day of purification. Neutrophils were primed with ultrapure O55:B5 LPS (Invivogen; 0.1 to 1 μg/ml), Pam3CSK4 (Invivogen; 0.1 to 1 μg/ml), Pam2CSK4 (Invivogen; 1 μg/ml), LTA (Invivogen; 1 μg/ml), IFN-γ (BioLegend; 10 to 100 ng/ml), or GM-CSF (Miltenyi Biotec; 10-100 ng/ml) for 4 hours and stimulated with nigericin (Invivogen; 5 μM) or TcdB (Abcam; 1 μg/ml) to activate the NLRP3 or Pyrin inflammasome, respectively. To activate the NLRC4 inflammasome, primed neutrophils were stimulated with a combination of LFn-Rod (Invivogen; 2 μg/ml) and PA protein (Merck; 2 μg/ml), centrifuged at 700*g* for 10 min at room temperature, and incubated at 37°C for 4 hours.

### LDH and ELISA

Cell lysis was quantified by measuring the amount of LDH released into the cell culture supernatant using a cytotoxicity detection kit (Sigma-Aldrich). The percentage of LDH release was calculated relative to 100% cell lysis in the untreated control. IL-1α (R&D Systems) and IL-1β (R&D Systems or Thermo Fisher Scientific) levels in cell-free supernatant were measured by enzyme-linked immunosorbent assay (ELISA) following the manufacturers’ protocols.

### Immunoblotting

Cell-free supernatants were precipitated using methanol and chloroform as previously described (*58*). Precipitated supernatant and cell extracts were resuspended in lysis buffer (2% SDS, 66 mM tris-Cl, pH 7.4, 10 mM dithiothreitol, NuPage LDS sample buffer; Thermo Fisher Scientific). Proteins were boiled at 95°C for 10 min and separated on 12 or 15% polyacrylamide gels and transferred onto nitrocellulose membrane using Transblot Turbo (Bio-Rad). Antibodies for immunoblot were against caspase-1 (AG-20B-0042-C100; 1:1000; Adipogen), GSDMD (ab209845; 1:1000; Abcam), IL-1β (AF-401-NA; 1:1000; R&D Systems), NLRP3 (Cryo-2; 1:1000; Adipogen), NLRC4 (ab201792, 1:1000; Abcam), Pyrin (ab214772; 1:1000; Abcam), ASC (ab309497; 1:1000; Abcam), α-tubulin (DM1A; 1:5000; Abcam or 2144; 1:5000; Cell Signaling Technology), and GAPDH (ab8245; 1:5000; Abcam). Nitrocellulouse membranes were developed using Clarity (Bio-Rad) or SuperSignal West Femto (Thermo Fisher Scientific) and imaged using the ChemiDoc Imaging System (Bio-Rad) or iBright imager (Thermo Fisher Scientific). Protein fold change was quantified using Image Lab (Bio-Rad).

### Scanning electron microscopy

A total of 4 × 10^5^ neutrophils were seeded on sterile glass slides. Neutrophils were primed with Pam3CSK4 (1 μg/ml) alone, coprimed with GM-CSF (100 ng/ml) and ultrapure Pam3CSK4 (1 μg/ml), or coprimed with GM-CSF (100 ng/ml) and ultrapure O55:B5 LPS (1 μg/ml) for 4 hours before stimulating with nigericin (5 μM) for 2 hours. Glass slides were pre-fixed with 2.5% glutaraldehyde for 2 hours, then post-fixed with 1% OsO_4_ for 1 hour, dehydrated in a graded ethanol series (25, 50, 75, 90, and 100%), dried using a critical point drier (LEICA EM CPD300), sputter coated with gold (LEICA EM ACE200), and examined at 10 kV using a scanning electron microscope (Quanta 650FEG, FEI).

### Flow cytometry

Single-cell suspension from bone marrow and spleen was prepared and red blood cells were lysed using ACK lysis buffer. Cells were stained with the following antibodies: α-CD45 (I3/2.3), α-CD11b (M1/70), α-Ly6G (clone 1A8), α-Ly6C (HK1.4), α-CD19 (6D5), and α-CD3 (17A2) and analyzed using the NovoCyte Penteon Flow Cytometer. Dead cells were identified and excluded using Zombie NIF or Zombie Yellow (BioLegend).

### Quantitative PCR

RNA was extracted from neutrophils using the RNeasy Mini kit (Qiagen) and reverse transcribed into cDNA using the GoScript Reverse Transcriptase kit (Promega). Quantitative PCR was performed using SYBR Green on a 7500 Real-Time PCR System (Applied Biosystem). Primer sequences are as follows: *Tlr2* (F: CCAGACACTGGGGGTAACATC; R: CGGATCGACTTTAGACTTTGGG), *Tlr4* (F: GGATTTATCCAGGTGTGAAATTG; R: GGGTTTCCTGTCAGTATCAAG), and *Hprt* (F: GCAGTACAGCCCCAAAATGG; R: AACAAAGTCTGGCCTGTATCCAA)*.*

### Statistical analyses

Data were analyzed with Prism 10 (GraphPad). Statistical significance was assessed using a *t* test for datasets with two groups, while a two-way analysis of variance (ANOVA) was applied to datasets involving more than two variables. Data were considered significant when *P ≤ 0.05, **P ≤ 0.01, ***P ≤ 0.001, or ****P ≤ 0.0001.
