# Supplementary material for: Threat assessment shapes neutrophil cell fate upon inflammasome activation
Source: Sci Adv. 2026 Jun 26;12(26):eaeb4830. doi: 10.1126/sciadv.aeb4830 (PMC13308611; doi:10.1126/sciadv.aeb4830)
Supplement: Supplementary file 1 — Figs. S1 to S8 [file sciadv.aeb4830_sm.pdf]

Science Advances
AAAS

Supplementary Materials for

Threat assessment shapes neutrophil cell fate upon inflammasome activation

See Jie Yow *et al.*

Corresponding author: Kaiwen W. Chen, Kaiwen.chen@nus.edu.sg

Sci. Adv. **12**, eaeb4830 (2026)
DOI: 10.1126/sciadv.aeb4830

This PDF file includes:

Figs. S1 to S8

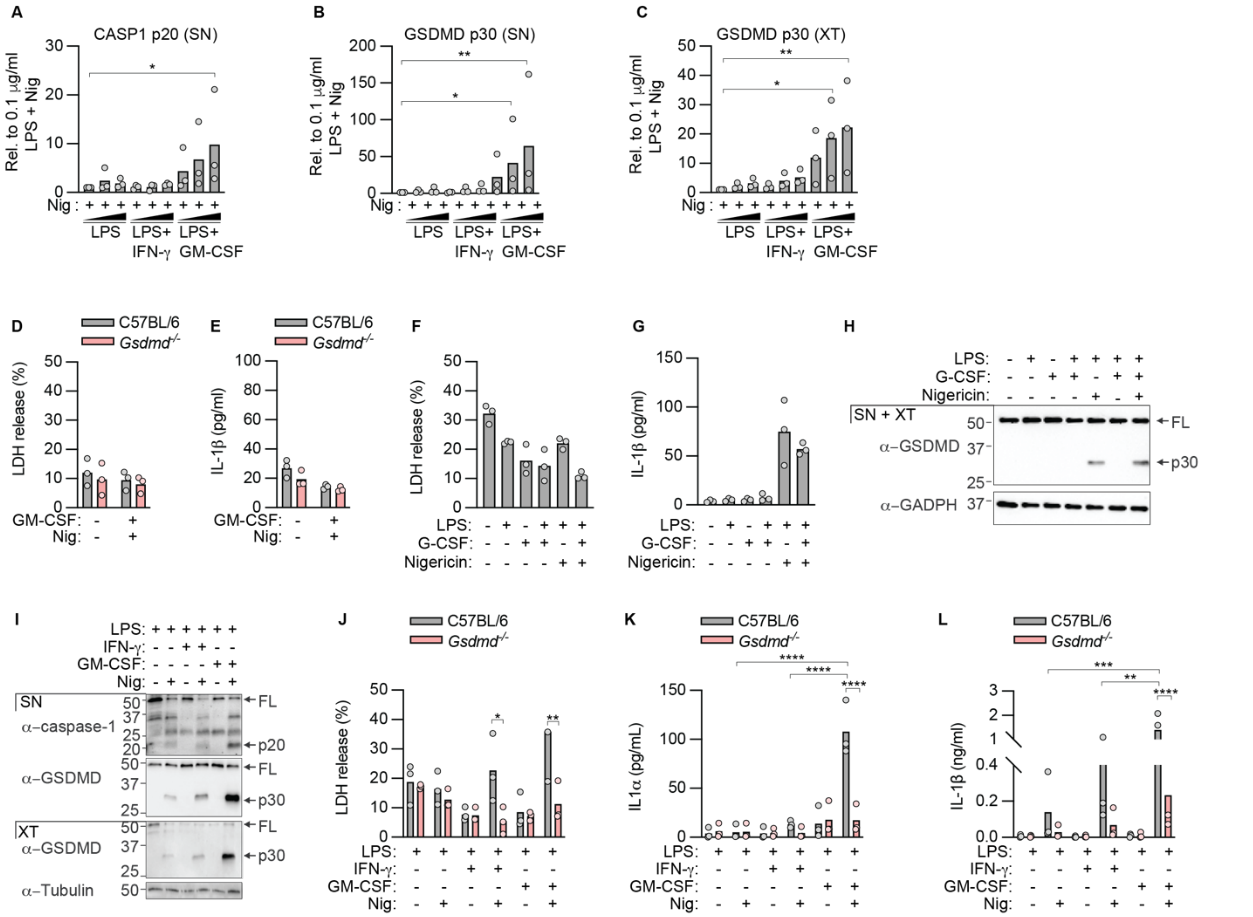

Figure S1. G-CSF does not sensitise LPS-primed neutrophils to pyroptosis. (A-C) Neutrophils were primed with increasing dose of ultrapure LPS (0.1, 0.5, 1μg/ml) in the presence or absence of IFN-γ (10, 50, 100μg/ml) or GM-CSF (10, 50, 100μg/ml) for 4 hours and stimulated with nigericin (5μM) for 2 hours. Relative amounts of **(A)** cleaved caspase-1 in the supernatant (SN) or **(B)** cleaved GSDMD in the SN or **(C)** cell extracts (XT) were quantified. **(D-H)** Neutrophils were primed with 100ng/ml GM-CSF, 1μg/ml LPS with and without 100ng/ml G-CSF for 4 hours and stimulated with nigericin (5μM) for 2 hours. **(D, F)** LDH release and **(E, G)** IL-1β secretion was quantified. **(H)** Precipitated supernatant (SN) and cell extracts (XT) were analysed by immunoblot. **(I-L)** Bone marrow neutrophils were purified by negative selection and primed with 1μg/ml LPS in the presence or absence of GM-CSF (100ng/ml) or IFN-γ (100ng/ml) and stimulated with nigericin (5μM) for 2 hours. **(I)** Precipitated supernatant (SN) and cell extracts (XT) were analysed by immunoblot. **(J)** LDH release and **(K-L)** IL-1 secretion was quantified. **(A-G, J-L)** Data represents mean value pooled from three independents. *$P < 0.05$, **$P < 0.01$, ***$P < 0.001$ and ****$P < 0.0001$.

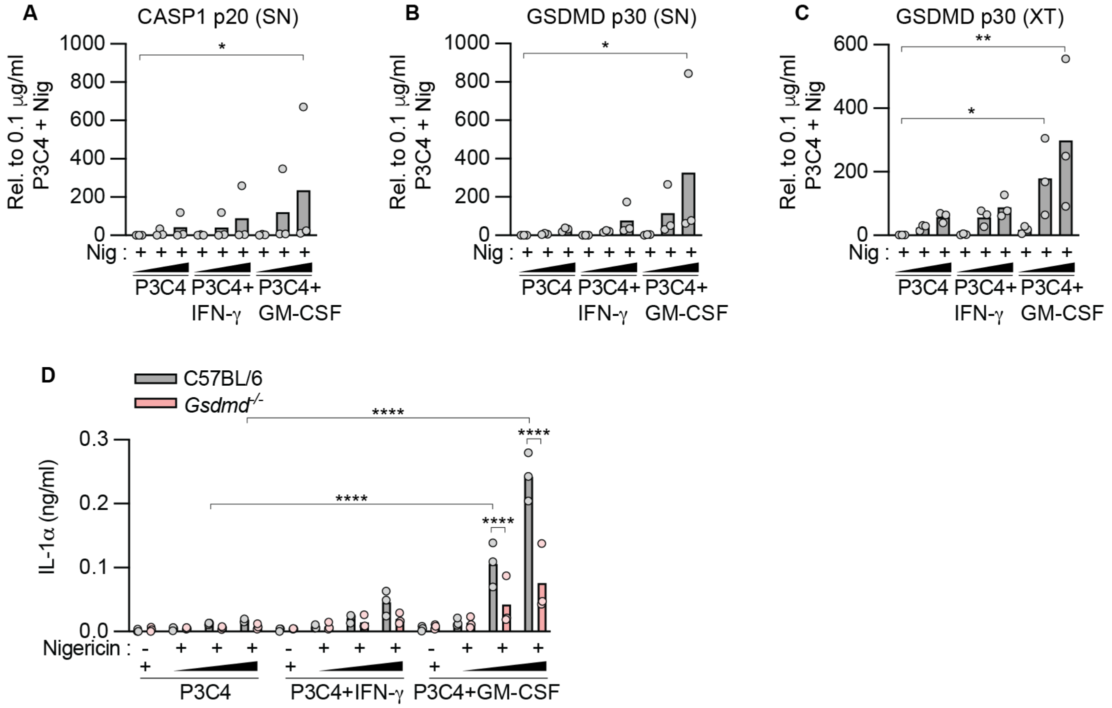

Figure S2. Pam3CSK4 priming alone is sufficient to license neutrophil pyroptosis upon NLRP3 activation. (A-D) Neutrophils were primed with increasing dose of Pam3CSK4 (P3C4; 0.1, 0.5, 1μg/ml) in the presence or absence of IFN-γ (10, 50, 100ng/ml) or GM-CSF (10, 50, 100ng/ml) for 4 hours and stimulated with nigericin (5μM) for 2 hours. Relative amounts of **(A)** cleaved caspase-1 in the supernatant (SN) or **(B)** cleaved GSDMD in the SN or **(C)** cell extracts (XT) were quantified. **(D)** IL-1α secretion was quantified. **(A-D)** Data represents mean value pooled from three independents. *$P < 0.05$, **$P < 0.01$ and ****$P < 0.0001$.

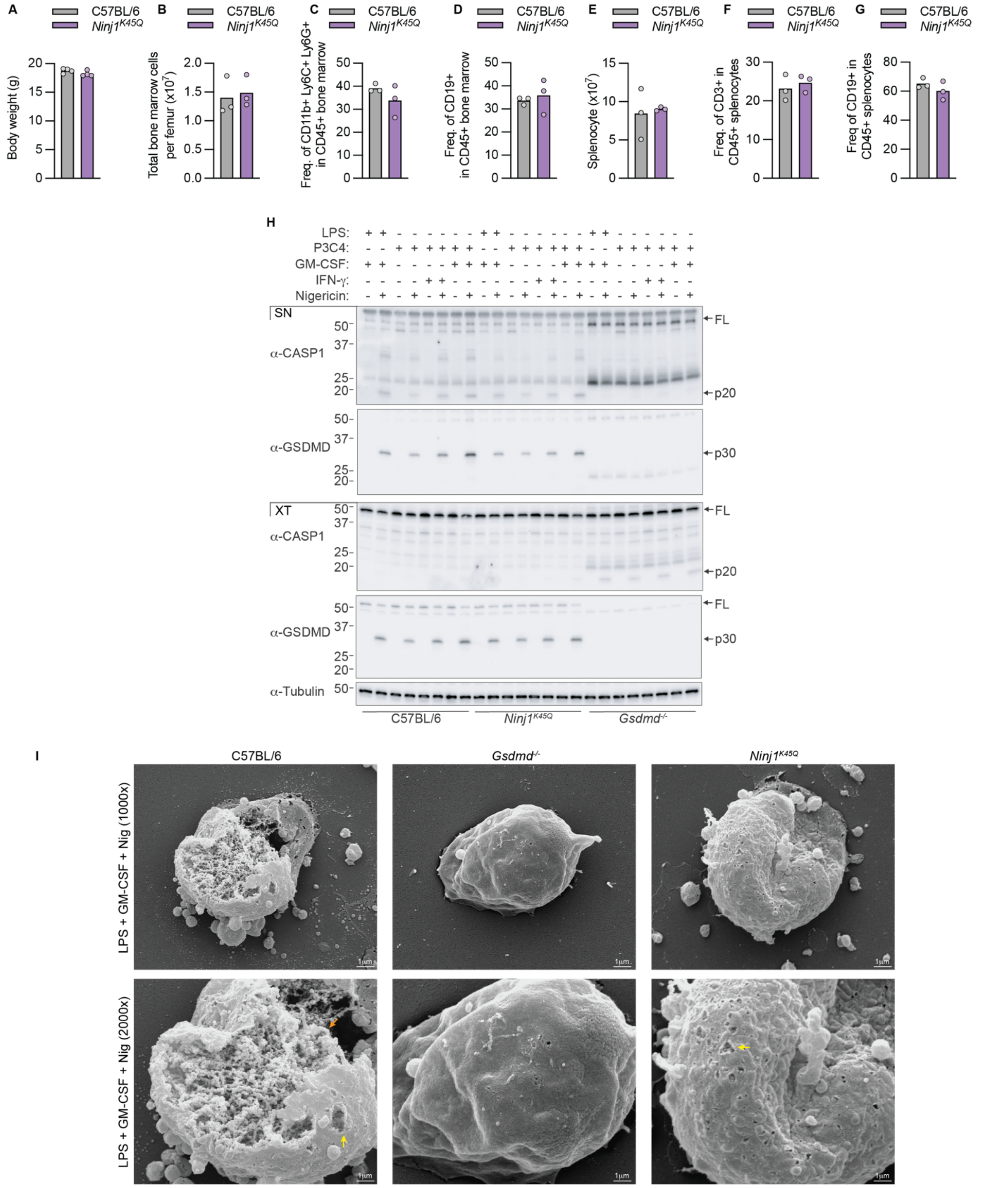

Figure S3. ***Ninj1*^{K45Q} mutation does not affect caspase-1 cleavage, GSDMD processing and IL-1a secretion.** **(A)** Body weight of 7-week-old male mice. **(B)** Total number of bone marrow cells isolated per femur in 8-week old male mice. Frequency of **(C)** $CD45^+CD11b^+Ly6C^+Ly6G^+$ neutrophils, **(D)** $CD45^+CD19^+$ B cells from the bone marrow of 8-week-old male mice. **(E)** Total number of splenocytes isolated from 8-week male mice. Frequency of **(F)** $CD45^+CD3^+$ T cells and **(G)** $CD45^+CD19^+$ B cells in the splenocytes of 8-week-old male mice. **(H-I)** Neutrophils were primed with ultrapure LPS (1μg/ml) or Pam3CSK4 (P3C4; 1μg/ml) in the presence or absence of

IFN-γ (100 ng/ml) or GM-CSF (100 ng/ml) for 4 hours and stimulated with nigericin (5μM) for 2 hours. **(H)** Precipitated supernatant (SN) and cell extracts (XT) were analysed by immunoblot. **(I)** Scanning electron microscopy images of nigericin-stimulated neutrophils. Orange arrow denotes large membrane pores (>1μM) while yellow arrows denote small membrane pores (<1μM). **(A-G)** Data represents mean value from 3-4 mice per genotype.

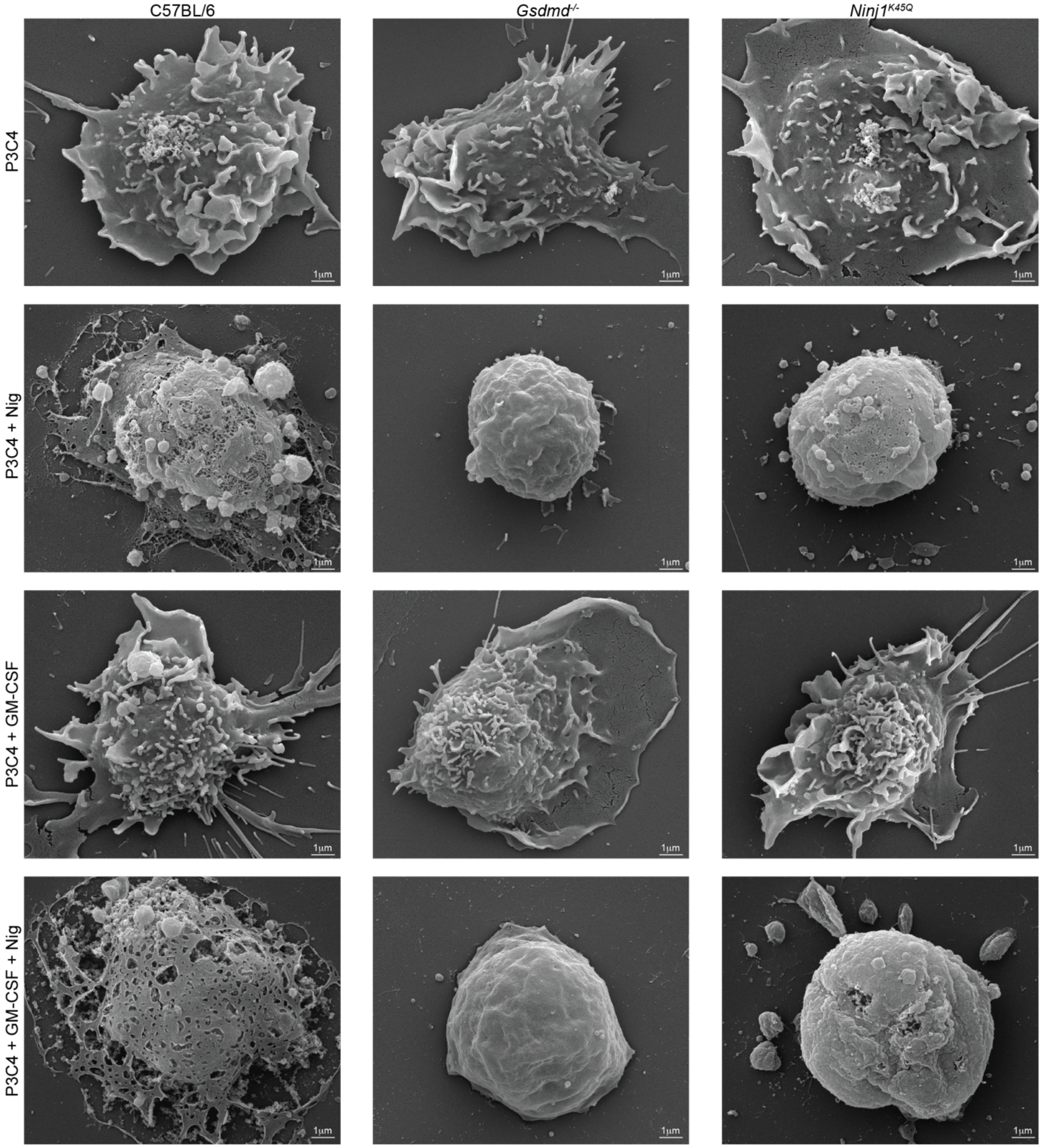

Figure S4. Pam3CSK4 or Pam3CSK4 and GM-CSF co-priming license neutrophils to undergo GSDMD and NINJ1-depenedent plasma membrane rupture upon nigericin stimulation. Neutrophils were primed with of Pam3CSK4 (1μg/ml) in the presence or absence of GM-CSF (100ng/ml) and stimulated with nigericin (5μM) for 2 hours and scanning electron microscopy was used to image these cells.

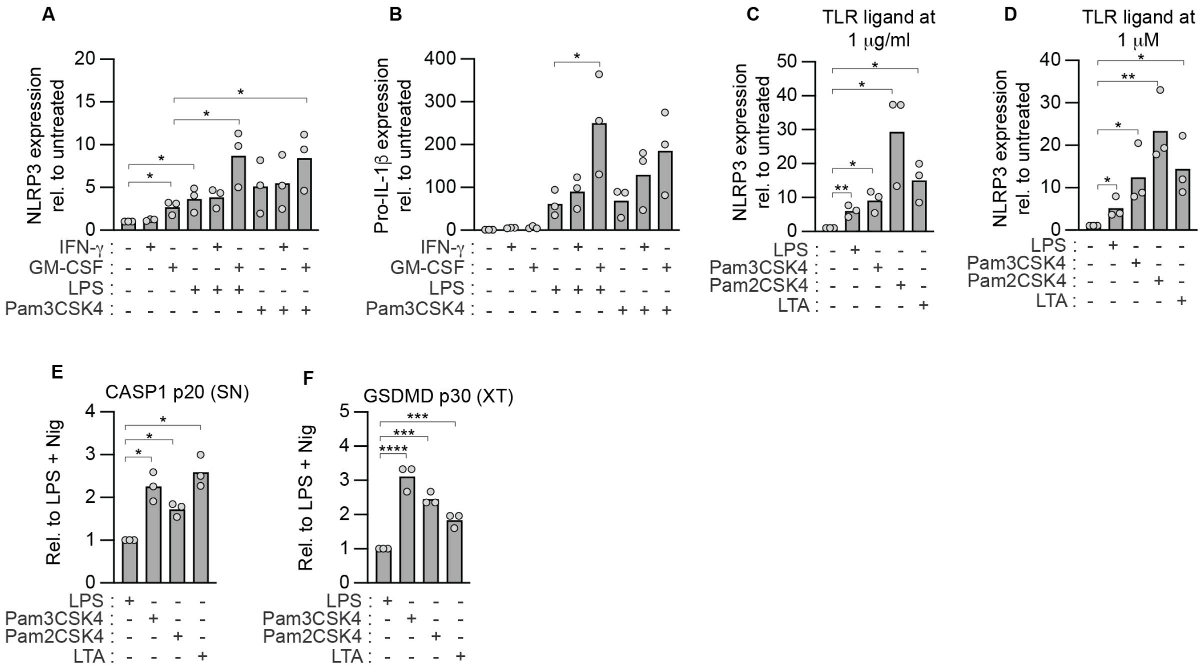

Figure S5. Neutrophil NLRP3 expression following various priming regime. (A-D) Neutrophils were primed with various combination of IFN-γ (100 ng/ml), GM-CSF (100 ng/ml), LPS (1μg/ml or 1μM), Pam3CSK4 (P3C4; 1μg/ml or 1μM), Pam2CSK4 (P2C4; 1μg/ml or 1μM) or LTA (1μg/ml or 1μM) for 4 hours and cell extracts were analysed by immunoblotting. Relative amounts of **(A, C-D)** NLRP3 and **(B)** pro-IL-1β in cell extracts (XT) were quantified. **(E-F)** Neutrophils were primed with LPS (1μg/ml or 1μM), Pam3CSK4 (P3C4; 1μg/ml or 1μM), Pam2CSK4 (P2C4; 1μg/ml or 1μM) or LTA (1μg/ml or 1μM) for for 4 hours and stimulated with nigericin (5μM) for 2 hours. Relative amounts of **(E)** cleaved caspase-1 in the supernatant and **(F)** cleaved GSDMD in cell extracts (XT) were quantified. **(A-F)** Data represents mean value pooled from three independents. **P* < 0.05, ***P* < 0.01 and *****P* < 0.0001.

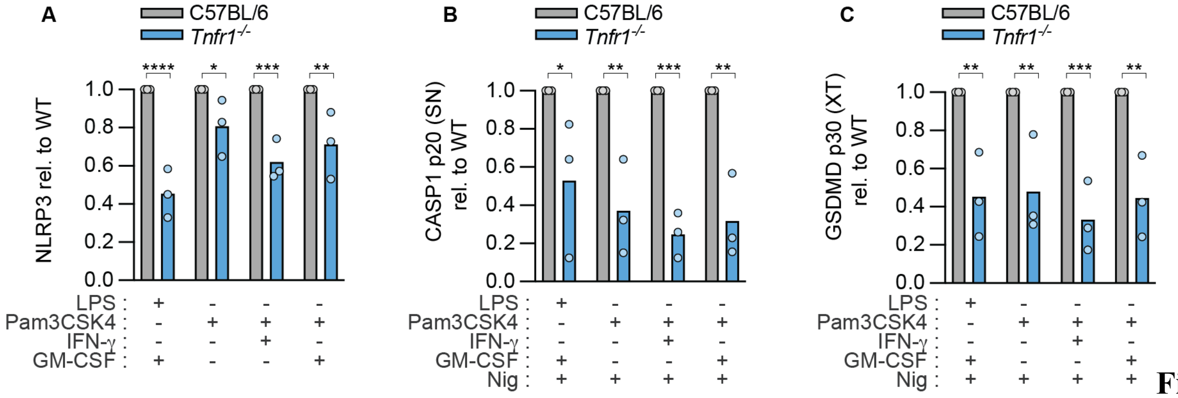

Figure S6. TNFR1 signalling promotes NLRP3 expression and nigericin-induced pyroptosis. (A) Neutrophils were primed with various combination of IFN-γ (100 ng/ml), GM-CSF (100 ng/ml), LPS (1µg/ml) or Pam3CSK4 (P3C4; 1µg/ml) for 4 hours and relative NLRP3 expression was analysed. **(B-C)** Neutrophils were primed with various combination of IFN-g (100 ng/ml), GM-CSF (100 ng/ml), LPS (1µg/ml) or Pam3CSK4 (P3C4; 1µg/ml) for 4 hours and stimulated with nigericin (5µM) for 2 hours. Relative amounts of **(B)** cleaved caspase-1 in the supernatant and **(C)** cleaved GSDMD in cell extracts (XT) were quantified. **(A-C)** Data represents mean value pooled from three independents. $*P < 0.05$, $**P < 0.01$ and $****P < 0.0001$.

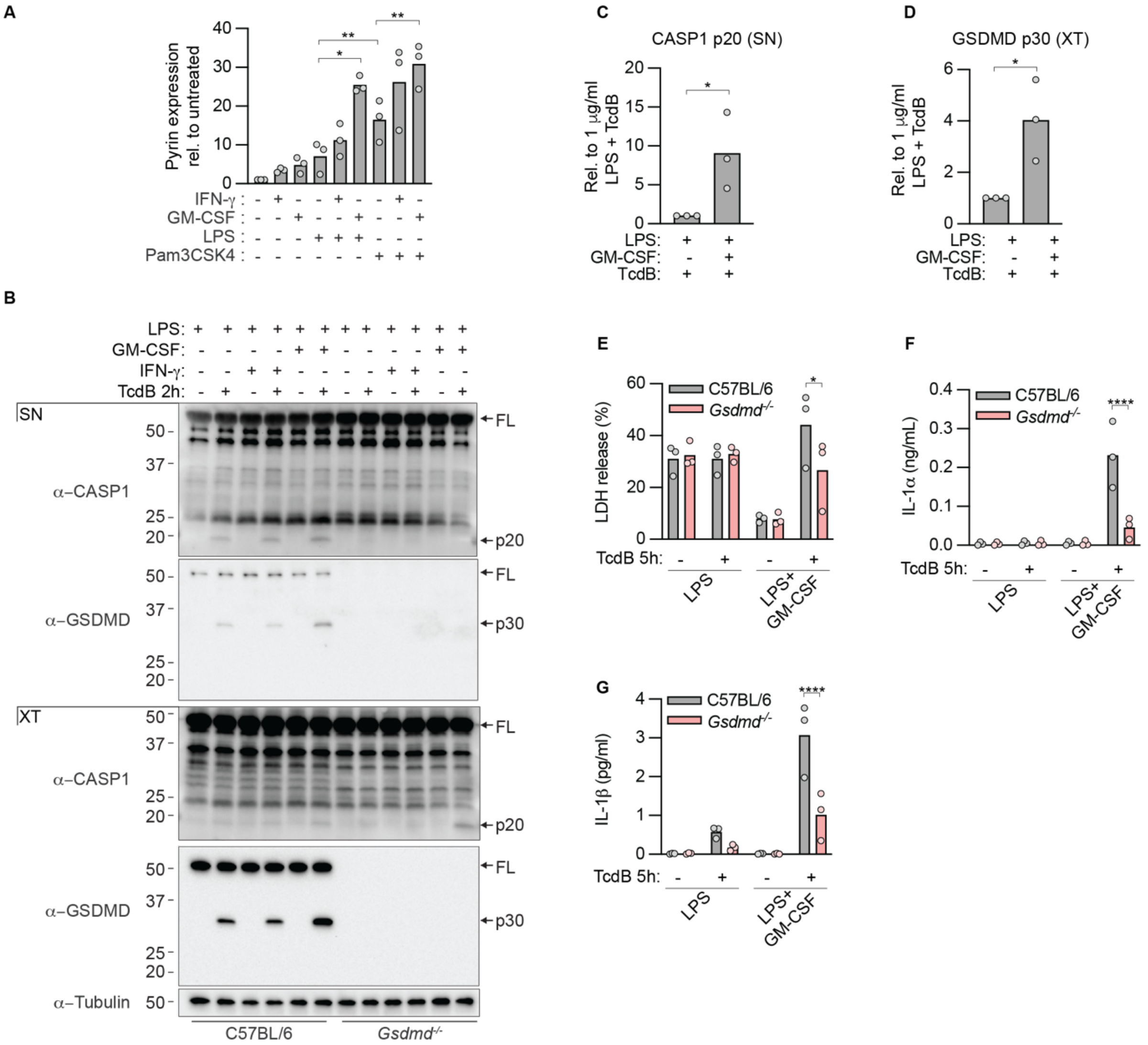

Figure S7. GM-CSF and LPS co-primed or Pam3CSK4 primed neutrophils are sensitive to Pyrin-dependent pyroptosis upon TcdB stimulation. (A) Neutrophils were primed with various combination of ultrapure LPS (1μg/ml) or Pam3CSK4 (P3C4; 1μg/ml) IFN-γ (100 ng/ml) or GM-CSF (100 ng/ml) for 4 hours and cell extracts were analysed by immunoblotting. **(B-G)** Neutrophils were primed with ultrapure LPS (1μg/ml) or Pam3CSK4 (P3C4; 1μg/ml) in the presence or absence of IFN-g (100 ng/ml) or GM-CSF (100 ng/ml) for 4 hours and stimulated with TcdB (1μg/ml) for **(B)** 2 hours or **(C-D)** 5 hours. **(B)** Precipitated supernatant (SN) and cell extracts (XT) were analysed by immunoblot. Relative amounts of **(C)** cleaved caspase-1 in the supernatant and **(D)** cleaved GSDMD in cell extracts (XT) were quantified. **(E-G)** Bone marrow neutrophils were purified by negative selection and primed with ultrapure LPS (1μg/ml) in the presence or absence of GM-CSF (100 ng/ml) for 4 hours, followed by TcdB (1μg/ml) stimulation for another 5 hours. **(E)** LDH release and **(F-G)** IL-1 secretion was quantified. **(A, C-G)** Data represents mean value pooled from three independents. *$P < 0.05$ and **$P < 0.01$.

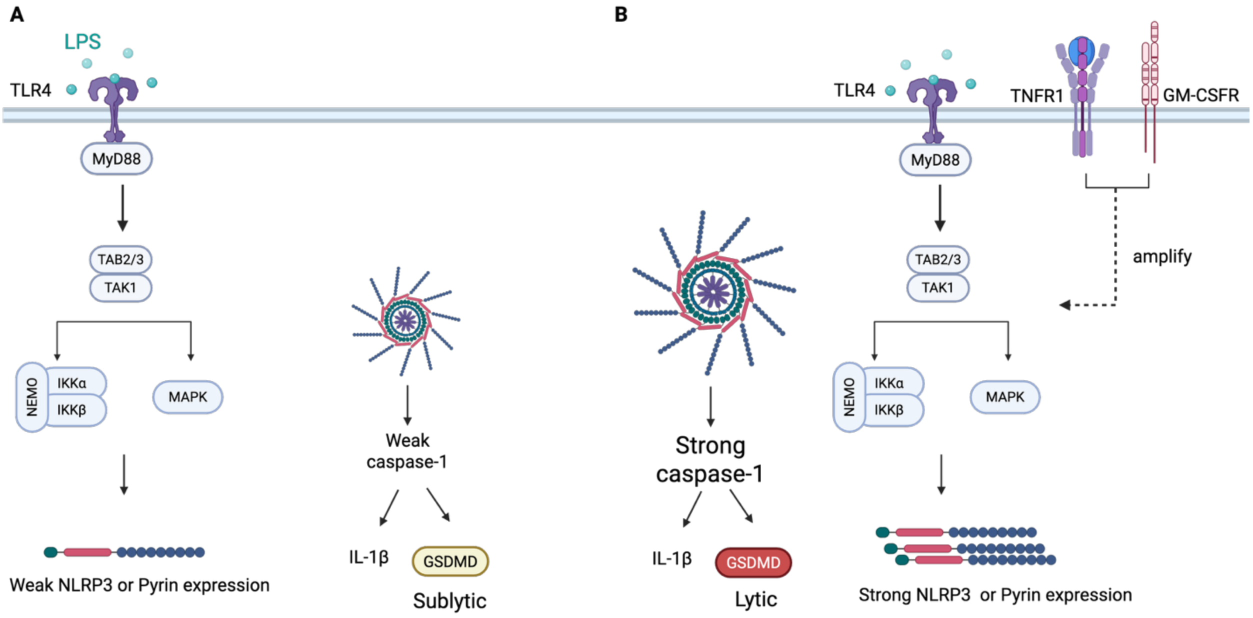

Figure S8. Summary model for mechanisms by which appropriate priming sensitises neutrophils to GSDMD-dependent pyroptosis. See discussion for more details.
